# A nationwide study of pathogens in hospitalized patients with lower respiratory tract infections by metagenomic next-generation sequencing

**DOI:** 10.1128/spectrum.01746-26

**Published:** 2026-09-23

**Authors:** Tian Gong, Qiong Liu, Yiqi Wan, Xuan Zhang, Chao Liu, Xiaojia Wang, Ling Cao, Jing Li, Liang Zhang, Biaoxian Li, Chensong Sun, Yaping Guo, Shupeng Yin, Xiumei Xu, Hao Chen, Zhiting Xie, Manli Gong, Haiyan Lin, Xiao Lei, Ting Zeng, Xinyi Ren, Shaomin Zou, Sufeng Zhang, Qifei Peng, Aling Gao, Jun Wang, Chengsheng Zhang

**Affiliations:** 1 Center for Molecular Diagnosis and Precision Medicine, The First Affiliated Hospital, Jiangxi Medical College, Nanchang University 47861, Nanchang, China; 2 Department of Medical Genetics, The First Affiliated Hospital, Jiangxi Medical College, Nanchang University 47861, Nanchang, China; 3 Jiangxi Provincial Center for Advanced Diagnostic Technology and Precision Medicine, The First Affiliated Hospital, Jiangxi Medical College, Nanchang University 47861, Nanchang, China; 4 Department of Medical Microbiology, School of Basic Medical Sciences, Jiangxi Medical College, Nanchang University 47861, Nanchang, China; 5 Medical Department, Hangzhou, Pan-omics Biotechnology Co., Ltd., Hangzhou, Zhejiang, China; 6 School of Public Health, Jiangxi Medical College, Nanchang University 47861, Nanchang, China; Children's National Hospital, George Washington University, Washington, DC, USA

**Keywords:** epidemiological survey, COVID-19, metagenomic next-generation sequencing, pathogen spectrum, lower respiratory tract infections

## Abstract

Lower respiratory tract infections remain a major cause of morbidity and mortality, yet timely etiologic diagnosis is difficult because of the breadth of possible pathogens and the limitations of conventional microbiological tests. We retrospectively analyzed 30,819 clinical specimens from 27,507 hospitalized patients with lower respiratory tract infections across 31 provincial-level regions in mainland China from April 2019 to May 2025 to characterize the pathogen spectrum detected by metagenomic sequencing. In total, 323 pathogens were identified, 33.74% of which were fastidious or unculturable microorganisms. The overall positivity rate was 80.12%, and 65.48% of positive specimens showed co-detections. The top 20, 50, 100, 150, and 200 pathogens accounted for 70.17%, 87.56%, 95.59%, 98.18%, and 99.26% of all reported pathogens, respectively. Pathogen distributions differed by age, season, geography, clinical department, and comorbidity, and several epidemiological shifts were observed across the pre-pandemic, pandemic, and post-restriction periods, including persistent declines in *Streptococcus pneumoniae* and *Chlamydia psittaci*. These findings demonstrate the breadth of organisms detected by metagenomic sequencing in routine practice and support its value for broad etiologic assessment of lower respiratory tract infections.

**IMPORTANCE:**

Lower respiratory tract infections are frequently managed before the etiologic agent is established because many bacteria, viruses, fungi, and other organisms can produce similar clinical syndromes, and conventional tests do not capture this diversity well. By analyzing more than 30,000 real-world specimens from hospitalized patients across mainland China, this study shows which organisms are most often detected by metagenomic sequencing, how frequently mixed detections occur, and how pathogen patterns vary by age, geography, season, clinical setting, and the 2019 coronavirus disease period. These findings clarify where broad-range sequencing can add value for microbiology, surveillance, and future targeted panel design.

## INTRODUCTION

Lower respiratory tract infections (LRTIs), including pneumonia and bronchiolitis, remain a major global cause of illness and death. In 2021, an estimated 344 million LRTI episodes and 2.18 million deaths occurred worldwide, while bacterial antimicrobial resistance was associated with 4.71 million deaths globally (1, 2). Accurate and timely etiologic diagnosis is therefore central to effective antimicrobial treatment, infection prevention, and public health surveillance. However, the clinical manifestations of LRTIs overlap across a diverse range of bacteria, fungi, viruses, and parasites, and conventional microbiological tests do not capture this diversity well (3–5). Culture and serology may have limited sensitivity for fastidious, unculturable, or previously treated organisms. Targeted nucleic acid amplification tests (NAATs), including thermal and isothermal amplification and CRISPR-Cas-assisted formats, are rapid and sensitive but require prespecified targets and therefore interrogate a finite panel (6). Sensor arrays and other emerging rapid platforms can improve analytical sensitivity and may support bacterial identification or antimicrobial susceptibility testing, but their organism coverage remains predefined, and clinical validation is still evolving (7). Diagnostic delay or misidentification may contribute to inappropriate antimicrobial exposure, disease progression, and missed opportunities for infection-control intervention.

Since an actionable diagnosis of neuroleptospirosis was reported in 2014 (8), unbiased metagenomic next-generation sequencing (mNGS) has increasingly been applied to infectious-disease diagnosis. It is a hypothesis-free, culture-independent method that detects microbial nucleic acids directly from clinical specimens and can identify rare or unexpected organisms represented in reference databases (9–11). mNGS has shown incremental diagnostic value for tuberculosis, viruses, anaerobic bacteria, and fungi, and has been used for etiologic assessment of LRTIs (11–13). Nevertheless, its broad analytical detection does not by itself establish causality, and large, geographically diverse real-world studies are needed to define organism distributions, co-detection patterns, and interpretation limits.

A previous epidemiological study evaluated associations of 14 common respiratory pathogens with age and disease severity in community-acquired pneumonia in China using RT-PCR and culture (14). Here, we retrospectively analyzed 30,819 mNGS reports from 27,507 hospitalized patients with LRTIs across mainland China from April 2019 to May 2025. We characterized detected organisms and compared their distributions across the pre-pandemic period (1 April to 31 December 2019), the COVID-19 pandemic and control period (1 January 2020 to 7 December 2022), and the post-restriction period (8 December 2022 to 31 May 2025). Our objective was to define the breadth and epidemiological distribution of mNGS-reported organisms and to generate hypotheses for surveillance and future targeted assay design.

## MATERIALS AND METHODS

### Patients and samples

The mNGS tests were ordered by clinicians as send-out tests to Matridx Biotechnology Co., Ltd. (Hangzhou, China). Demographic data, routine test results, and preliminary diagnoses were partially available from test requisition forms (TRFs), on which these fields were optional. Each patient or legal guardian consented to secondary use of de-identified testing data for research and development. We searched the database for records with a preliminary diagnosis of pneumonia or community-acquired pneumonia and retrieved reports from 27,507 patients. We retained respiratory specimens and peripheral blood, consolidated repeat analytical reports generated from the same specimen, and excluded report-level taxa that were not considered plausible respiratory pathogens after review of the submitted diagnosis, specimen type, published respiratory-pathogen literature, and the laboratory’s clinical reporting framework (15, 16). The excluded report-level taxa included organisms assigned to *Eubacterium*, *Campylobacter*, *Salmonella*, or *Aeromonas*. This filter was intended to reduce incidental gastrointestinal, oral, or environmental signals, and it does not imply that every species in these genera is incapable of causing rare pulmonary infection. It is possible that this may have excluded uncommon true infections and is acknowledged as a study limitation. Peripheral blood was retained because plasma microbial cell-free DNA may capture systemic or bloodstream involvement in severe pneumonia and can provide diagnostic information when a lower-respiratory specimen is unobtainable (9, 12).

### Preparation of internal controls

The internal-control (IC) DNA was synthesized, amplified using PrimeSTAR HS DNA polymerase (Takara, R044), purified with magnetic beads (Matridx, MD012), and quantified using the Qubit dsDNA HS assay (Thermo Fisher Scientific, Q32854). For plasma libraries, IC DNA was added after nucleic acid extraction at 0.02 ng/μL (17). The spike-in was used to monitor inhibition, library construction, sequencing yield, and downstream bioinformatic recovery in low-biomass cell-free DNA libraries. Because it was added after extraction, it did not assess extraction efficiency. An exogenous post-extraction IC was not added to non-plasma specimens, for which total nucleic-acid yield and run-level positive and negative controls were used for quality assessment.

### Library preparation and sequencing

Nucleic acids were extracted automatically; non-plasma samples underwent enzymatic fragmentation, followed by end repair, A-tailing, adaptor ligation, and library amplification as previously described (18). Libraries were quantified by real-time PCR (KAPA) and sequenced on an Illumina NextSeq 550Dx using a 75-cycle rapid kit, targeting approximately 20 million 50 bp single-end reads per sample. Reads failing instrument quality filters, low-quality reads, adapter sequences, low-complexity reads, and short reads were removed. Human reads were subtracted by alignment to GRCh38.p13. Remaining reads were aligned for taxonomic assignment against NCBI nt, GenBank, and an in-house curated microbial whole-genome reference collection. The curated collection was assembled from public reference genomes, de-duplicated, taxonomically annotated, and periodically reviewed to remove low-quality or ambiguously classified sequences. Species-level read counts and reads per million (RPM) were generated. Each sequencing run included a negative control containing fragmented human DNA and a positive control containing defined synthetic DNA or RNA fragments, including *Bacillus brevis*, *Candida albicans*, and influenza A virus. Run acceptance required the expected organisms in the positive control, no unexpected high-level signal in the negative control, and sample-level library and base-quality thresholds described below. The analytical workflow was specimen receipt and nucleic-acid extraction, plasma IC addition, library construction and qPCR quantification, NextSeq sequencing, read-quality filtering and human-read subtraction, microbial alignment and taxonomic assignment, control-based background filtering, and clinical report generation.

### Pathogen reporting criteria

A microbial taxon was eligible for reporting when (i) the sample passed quality thresholds (library concentration > 50 pM, Q20 > 85%, and Q30 > 80%); and (ii) the taxon was absent from the negative control in the same run or its RPM ratio, RPMsample/RPMnegative control, was at least 5. This empirically derived cutoff was based on prior multicenter and laboratory studies intended to reduce reagent and environmental background (19–22). Taxonomic assignments with close genomic similarity were interpreted cautiously; the potential for misassignment, particularly between *Streptococcus mitis* and *Streptococcus pneumoniae*, was considered during interpretation.

### Statistical analysis

Analyses were performed using SPSS version 25.0 (IBM). Categorical variables are presented as counts and percentages and were compared using Pearson’s chi-square test; Fisher’s exact test was used when expected cell counts were <5. Continuous variables were assessed for normality using the Shapiro-Wilk test and inspection of Q-Q plots. Normally distributed variables are summarized as mean (standard deviation) and compared using parametric tests. Non-normally distributed variables are summarized as median (interquartile range) and compared using the Kruskal-Wallis test or Wilcoxon signed-rank test, as appropriate. For key proportions, 95% confidence intervals (CIs) were calculated using the Wilson method. Between-period effects are reported as absolute risk differences with 95% CIs. For exploratory families of pathogen-by-subgroup comparisons, false-discovery rate control by the Benjamini-Hochberg procedure was specified. Where adjusted results could not be reconstructed from the archived report-level export, findings are described as exploratory and interpreted from effect estimates and CIs rather than isolated *P* values. All tests were two-sided; *P* < 0.05 was considered statistically significant after prespecified multiplicity correction where applicable.

## RESULTS

### Characteristics of the enrolled patients

**Fig 1:**
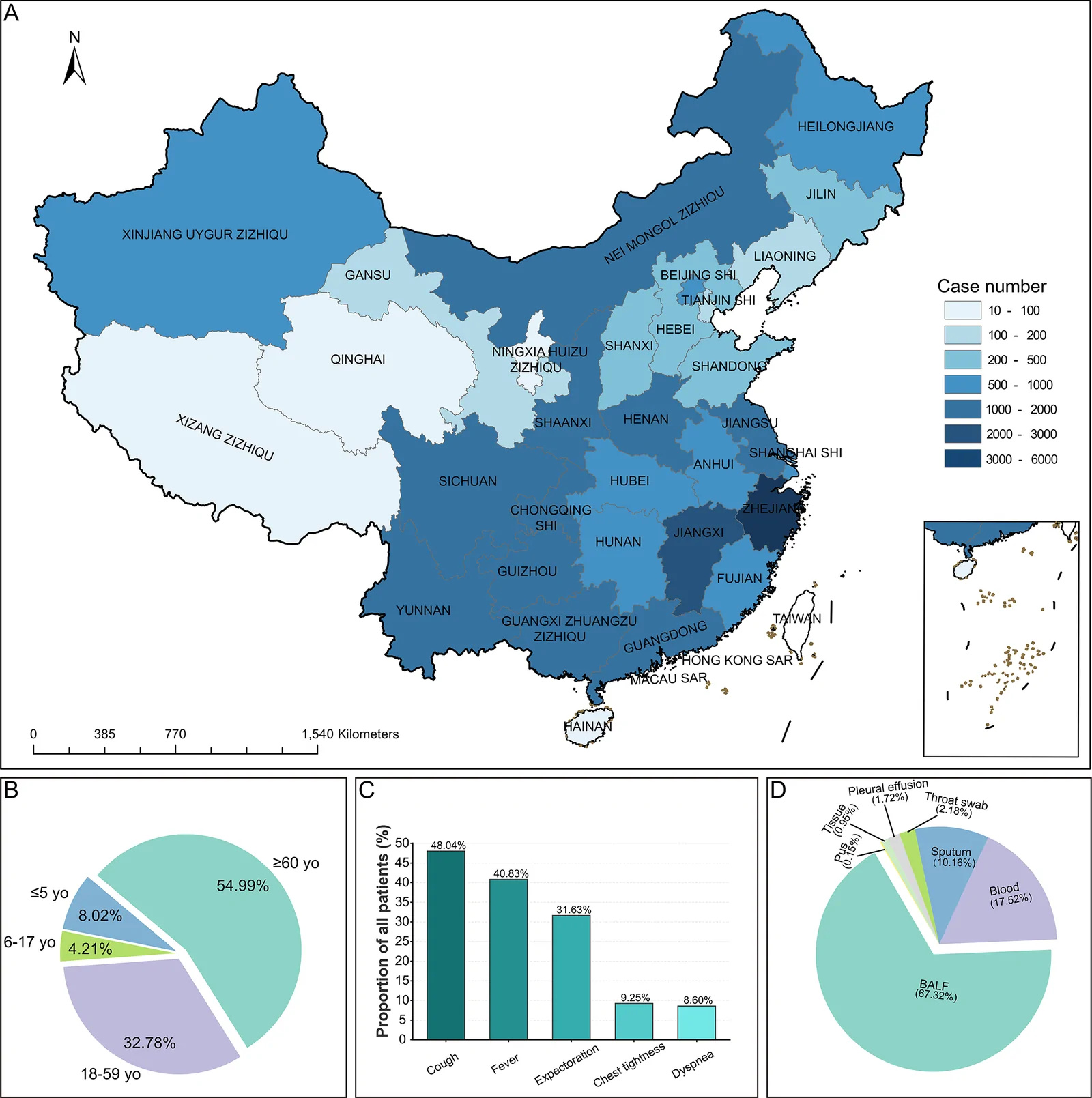
Origins and characteristics of the study cases. (**A**) Distribution of the source of mNGS specimens in the mainland of China from April 2019 to May 2025. The darker the color of each province, the larger the sample size. (**B**) Age group distribution. (**C**) Proportions of different clinical symptoms among all cases. (**D**) Proportions of different sample types.

In this study, we retrospectively analyzed 30,819 specimens from 27,507 patients who were hospitalized due to pneumonia. The clinicians ordered mNGS tests for these patients, and samples were transported to clinical laboratories for testing. The patients were from the tertiary hospitals located in 31 provinces, autonomous regions, and municipalities in China, and the data were collected from April 2019 to May 2025 (Fig. 1A). The median age was 56.0 years (0–107 years, 54.75% of the patients were over 60 years) (Fig. 1B), including 18,317 males (66.59%) and 9,190 females (33.41%). The primary clinical manifestations were cough (48.04%, 13,214/27,507), fever (40.83%, 11,231/27,507), and expectoration (31.63%, 8,700/27,507), which were consistent with LRTIs (Fig. 1C). The samples included in this study were diverse, with bronchoalveolar lavage fluid (BALF) being the most common (67.32%), followed by peripheral blood (17.52%), sputum (10.16%), throat swab (2.18%), pleural effusion (1.71%), lung tissue (0.95%), and specimens labeled as pus (0.15%; 46/30,819) (Fig. 1D). The pus category comprised clinician-submitted purulent specimens linked to an LRTI or pneumonia diagnosis on the TRF. The exact anatomic source (e.g., lung-abscess drainage versus another intrathoracic suppurative collection) was not consistently captured, so these specimens could not be subclassified further.

### The pathogens detected by mNGS

**Fig 2:**
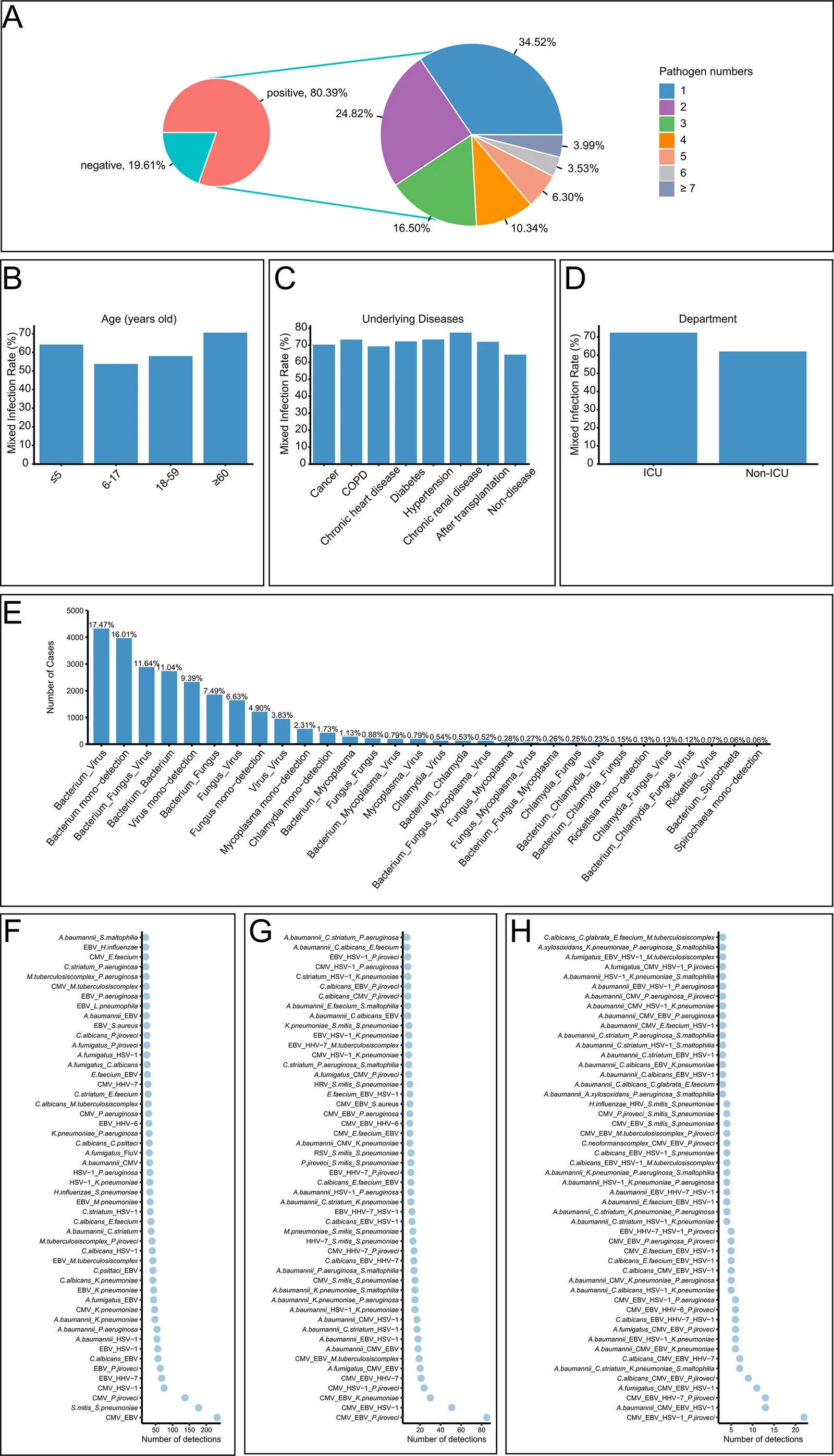
Co-detection of pathogens at various levels in positive reports. (**A**) The proportion of samples with and without detected pathogens, and the proportion of samples with different quantities of pathogens detected. (**B**) The proportion of multiple infections in various age groups. (**C**) The proportion of multiple infections with underlying diseases. (**D**) The proportion of multiple infections in ICU or non-ICU. (**E**) Statistics on co-detection following pathogen classification. Co-detection results with fewer than 10 positive samples were omitted. (**F**) Pathogen co-detection statistics were collected in samples with two detected pathogens. (**G**) Pathogen co-detection statistics were collected in samples with three detected pathogens. (**H**) Pathogen co-detection statistics were collected in samples with four detected pathogens.

In this study, we detected the pathogens by mNGS and revealed that the overall positive detection rate of all samples was 80.12% (24,774/30,819). A total of 323 pathogens were identified from these samples (Table S1), of which bacteria represented the most prevalent category (*n* = 30,968, 48.13%), followed by viruses (*n* = 20,119, 31.27%) and fungi (*n* = 10,508, 16.33%). We also found that 65.48% (16,221/24,774) of specimens with positive mNGS findings exhibited co-detections (Fig. 2A), which was more common in children (≤5 years), elderly patients (≥60 years), and patients in the intensive care unit (ICU) (Fig. 2B through D).

No significant differences were found in the proportion of patients with different underlying diseases and co-detections. The most frequently occurring co-detections were bacterial-viral (17.47%), followed by bacterial-fungal-viral (11.64%), bacterial-bacterial (11.04%), bacterial-fungal (7.49%), fungal-viral (6.63%), and viral-viral (3.83%) (Fig. 2E). Further analysis revealed that CMV and Epstein-Barr virus (EBV), *Streptococcus mitis *and *Streptococcus pneumoniae*, CMV, and *Pneumocystis jirovecii* were the most common dual-detections, while CMV, EBV and *P. jirovecii* were the most common triple-detections (Fig. 2F through H).

**Fig 3:**
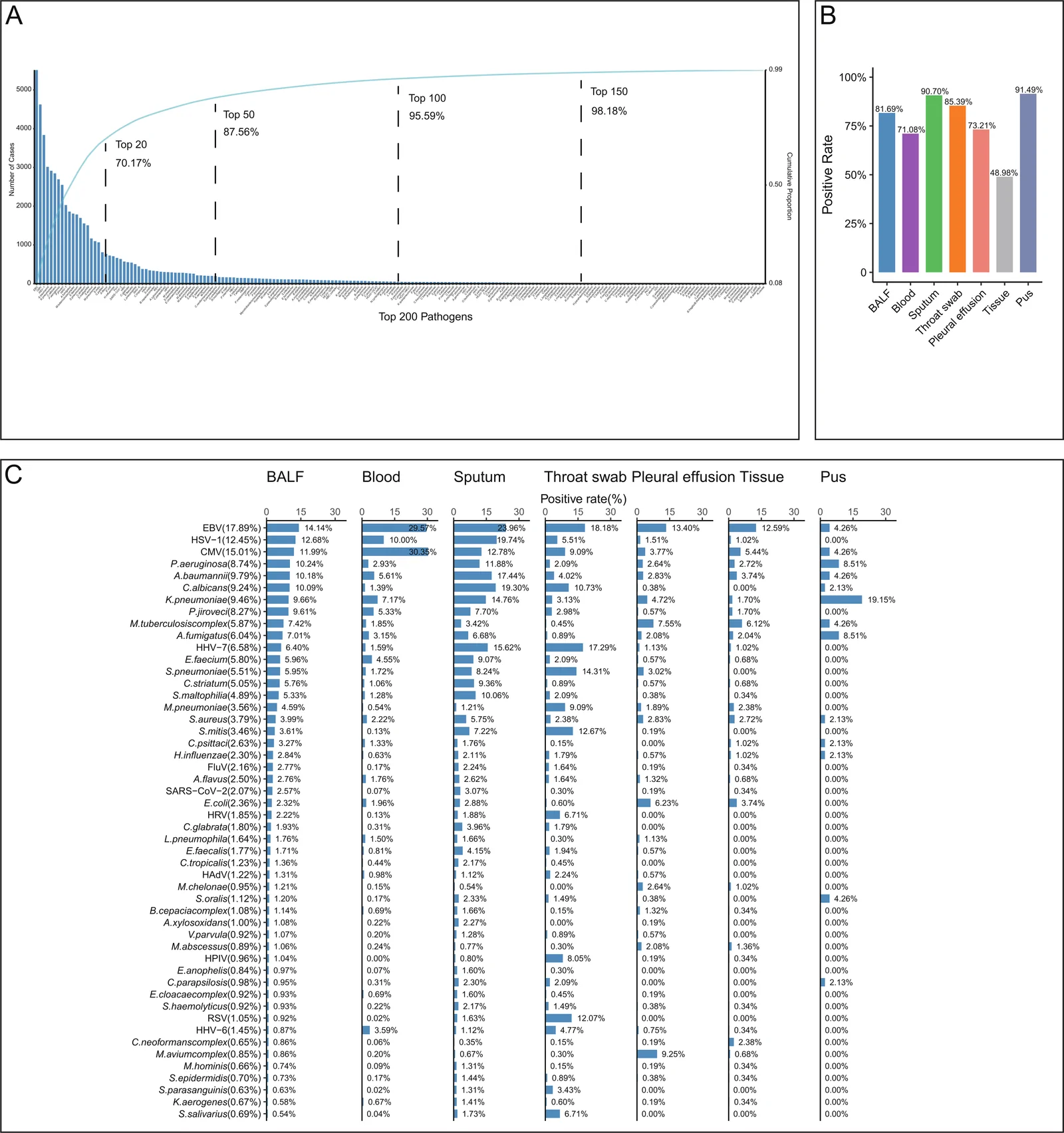
Pathogen spectrum characteristics of various sample types. (**A**) The top 50 pathogens observed in reported cases between April 2019 and July 2023. (**B**) Positive rate of various sample types. (**C**) Pathogen detection status for various sample types. The percentage in parentheses = The number of detections of the pathogen/Total number of positive samples. The percentage on the right side of the bar chart = The number of detections of the pathogen/The number of times each pathogen was detected in the sample/the number of corresponding sample types.

As illustrated in Fig. 3A, we ranked the frequency of the pathogens and found that the top 20, 50, 100, 150, and 200 accounted for 70.17%, 87.56%, 95.59%, 98.18%, and 99.26% of the reported pathogens, respectively. Among the top 50 pathogens (Table 1), the most frequently detected of bacteria was *Acinetobacter baumannii* (accounting for 9.79%, 3,016/30,819), followed by *Klebsiella pneumoniae* (9.46%, 2,914/30,819), *Pseudomonas aeruginosa* (8.74%, 2,694/30,819), *Mycobacterium tuberculosis* complex (5.87%, 1,809/30,819), and *Enterococcus faecium* (5.80%, 1,786/30,819). For viruses, the most commonly detected were herpesviruses, including EBV (accounting for 17.89%, 5,513/30,819), CMV (15.01%, 4,625/30,819), and HSV-1 (12.45%, 3,837/30,819). EBV and CMV were also commonly detected in blood (59.92%), sputum (36.74%), throat swab (27.27%), and BALF samples (26.13%) (Fig. 3C). In addition, respiratory viruses that are known to be associated with community-acquired pneumonia were also detected, such as severe acute respiratory syndrome coronavirus 2 (SARS-CoV-2, 2.07%, 637/30,819), human rhinovirus (HRV, 1.85%, 571/30,819), human adenovirus (HAdV, 1.22%, 377/30,819), respiratory syncytial virus (RSV, 1.05%, 323/30,819), and human parainfluenza virus (HPIV, 0.96%, 297/30,819). As for fungi, *Candida albicans* accounted for 9.24% (2,847/30,819) of all pathogens reported, followed by *P. jirovecii* (8.27%, 2,550/30,819) and *Aspergillus fumigatus* (6.04%, 1,860/30,819). The positive rates of sputum, throat swab, and BALF samples were 90.70%, 85.39%, and 81.69%, respectively (Fig. 3B and C).

**TABLE 1:** Top 50 pathogens detected from April 2019 to May 2025

Taxon name	Abbreviation	No. detected	Positive rate (%)
Bacterium			
*Acinetobacter baumannii*	*A. baumannii*	3,016	9.79
*Klebsiella pneumoniae*	*K. pneumoniae*	2,914	9.46
*Pseudomonas aeruginosa*	*P. aeruginosa*	2,694	8.74
*Mycobacterium tuberculosis* complex	*M. tuberculosis* complex	1,809	5.87
*Enterococcus faecium*	*E. faecium*	1,786	5.8
*Streptococcus pneumoniae*	*S. pneumoniae*	1,697	5.51
*Corynebacterium striatum*	*C. striatum*	1,555	5.05
*Stenotrophomonas maltophilia*	*S. maltophilia*	1,506	4.89
*Staphylococcus aureus*	*S. aureus*	1,167	3.79
*Streptococcus mitis*	*S. mitis*	1,067	3.46
*Escherichia coli*	*E. coli*	726	2.36
*Haemophilus influenzae*	*H. influenzae*	709	2.3
*Enterococcus faecalis*	*E. faecalis*	544	1.77
*Legionella pneumophila*	*L. pneumophila*	506	1.64
*Streptococcus oralis*	*S. oralis*	344	1.12
*Burkholderia cepacia* complex	*B. cepacia* complex	334	1.08
*Achromobacter xylosoxidans*	*A. xylosoxidans*	308	1
*Mycobacterium chelonae*	*M. chelonae*	292	0.95
*Staphylococcus haemolyticus*	*S. haemolyticus*	285	0.92
*Enterobacter cloacae* complex	*E. cloacae* complex	283	0.92
*Veillonella parvula*	*V. parvula*	282	0.92
*Mycobacterium abscessus*	*M. abscessus*	273	0.89
*Mycobacterium avium* complex	*M. avium* complex	263	0.85
*Elizabethkingia anophelis*	*E. anophelis*	258	0.84
*Staphylococcus epidermidis*	*S. epidermidis*	215	0.7
*Streptococcus salivarius*	*S. salivarius*	214	0.69
*Klebsiella aerogenes*	*K. aerogenes*	207	0.67
*Streptococcus parasanguinis*	*S. parasanguinis*	195	0.63
Virus			
Epstein-Barr virus	EBV	5,513	17.89
Cytomegalovirus	CMV	4,625	15.01
Human alphaherpesvirus 1	HSV-1	3,837	12.45
Human betaherpesvirus 7	HHV-7	2,028	6.58
Influenza virus	FluV	666	2.16
Severe acute respiratory syndrome coronavirus 2	SARS-CoV-2	637	2.07
Human rhinovirus	HRV	571	1.85
Human betaherpesvirus 6	HHV-6	447	1.45
Human adenovirus	HAdV	377	1.22
Respiratory syncytial virus	RSV	323	1.05
Human parainfluenza virus	HPIV	297	0.96
Fungus			
*Candida albicans*	*C. albicans*	2,847	9.24
*Pneumocystis jirovecii*	*P. jirovecii*	2,550	8.27
*Aspergillus fumigatus*	*A. fumigatus*	1,860	6.04
*Aspergillus flavus*	*A. flavus*	769	2.5
*Candida glabrata*	*C. glabrata*	554	1.8
*Candida tropicalis*	*C. tropicalis*	378	1.23
*Candida parapsilosis*	*C. parapsilosis*	301	0.98
*Cryptococcus neoformans* species complex	*C. neoformans* complex	201	0.65
Mycoplasma			
*Mycoplasma pneumoniae*	*M. pneumoniae*	1,098	3.56
*Mycoplasma hominis*	*M. hominis*	202	0.66
Chlamydia			
*Chlamydia psittaci*	*C. psittaci*	811	2.63

**Fig 4:**
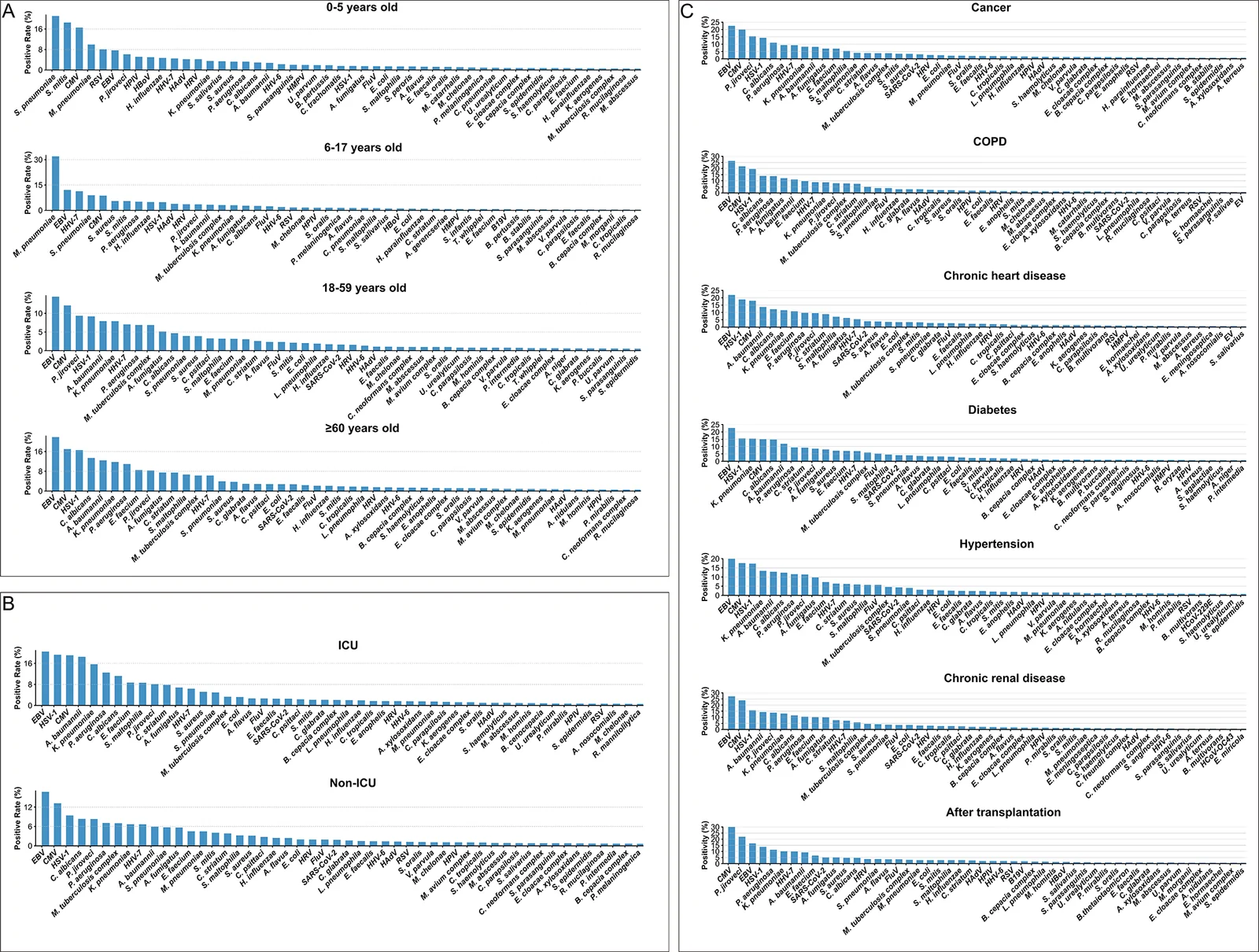
Detection of pathogens in different groups. Detection rate (%) = Number of detected pathogens in the group/Total number of people in the group. Panel **A** showed the groups of various ages; panel **B** showed the groups of ICU or non-ICU; panel **C** showed the groups of underlying diseases. ICU, intensive care unit; COPD, chronic obstructive pulmonary disease.

We then analyzed the top 50 pathogens according to age. Young children (≤5 years) displayed a higher incidence rate of *S. pneumoniae*, *S. mitis*, CMV, *Mycoplasma pneumoniae*, RSV, and EBV (Fig. 4A), whereas *M. pneumoniae* was primarily detected in children aged 6 to 17 years (Fig. 4A). In patients over 60 years, EBV, CMV, HSV-1, and *Chlamydia psittaci* were prevalent. Moreover, pathogens associated with nosocomial infections, such as *A. baumannii*, *K. pneumoniae*, *P. aeruginosa*, *C. albicans*, and *A. fumigatus*, were more predominant in elderly patients (Fig. 4A). We also compared the pathogen frequency according to the hospital departments. As expected, *A. baumannii* and *K. pneumoniae* were more frequently detected in ICU (Fig. 4B). Additionally, *P. jirovecii* was more common in patients with cancers or transplantations (Fig. 4C).

**Fig 5:**
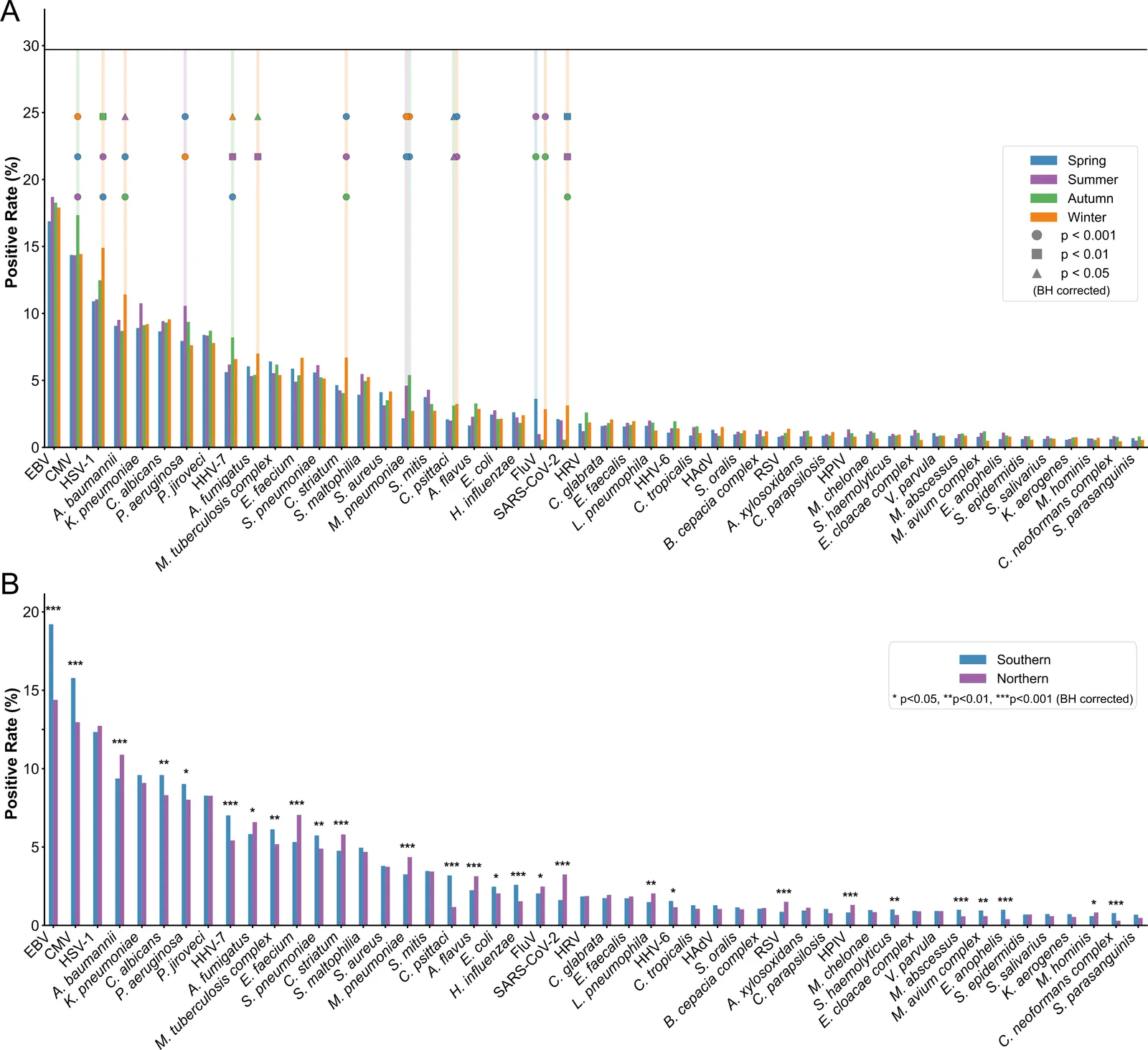
Seasonal and geographic prevalence of detected pathogens. (**A**) The detection rate of the top 50 pathogens in four seasons. Detection rate (%) = Number of detected pathogens in each season/Total sample size in that season × 100%. Pairwise comparisons of pathogen detection rates between different seasons were performed to specifically assess whether seasons with higher detection rates were statistically significantly higher than those with lower rates, and the Benjamini-Hochberg (BH) method was applied to correct for the false discovery rate (FDR), thereby mitigating the risk of type I errors associated with multiple comparisons. (**B**) The detection rate of the top 50 pathogens in North and South China. Detection rate (%) = Number of detected pathogens in the north and south/Total sample size in the north and south × 100%. Pairwise statistical analysis was conducted to compare pathogen detection rates between North China and South China, with a specific focus on determining whether the region with a higher detection rate was statistically significantly higher than the other, and the BH correction was used to adjust the FDR, ensuring the validity and reliability of results in the context of multiple comparison testing.

Next, we analyzed the seasonality of respiratory pathogens (Fig. 5A). Influenza viruses were dominant in the spring and winter while *P. aeruginosa* showed a higher detection rate in the summer. CMV and HHV-7 were more prevalent in the fall and HSV-1, *A. baumannii*, *A. fumigatus*, *Corynebacterium striatum,* and SARS-CoV-2 were found to be more prevalent in the winter. *M. pneumoniae* was more prevalent in the summer and fall, while *C. psittaci* was mostly detected in the autumn and winter.

As shown in Fig. 5B, the positive rates of the top pathogens were also analyzed by geographical regions (north versus south). Herpesviruses (such as EBV, CMV, HHV-6, HHV-7) and other pathogens (such as *P. aeruginosa*, *M. tuberculosis* complex, *S. pneumoniae*, *C. psittaci,* and *Haemophilus influenzae*) were more prevalent in the south, whereas *A. baumannii*, *M. pneumoniae*, *E. faecium*, *C. striatum*, and the majority of respiratory viruses (such as SARS-CoV-2, influenza virus, RSV, and HPIV) were more frequently detected in the north (Fig. 5B).

### Atypical and difficult-to-diagnose pathogens

**Fig 6:**
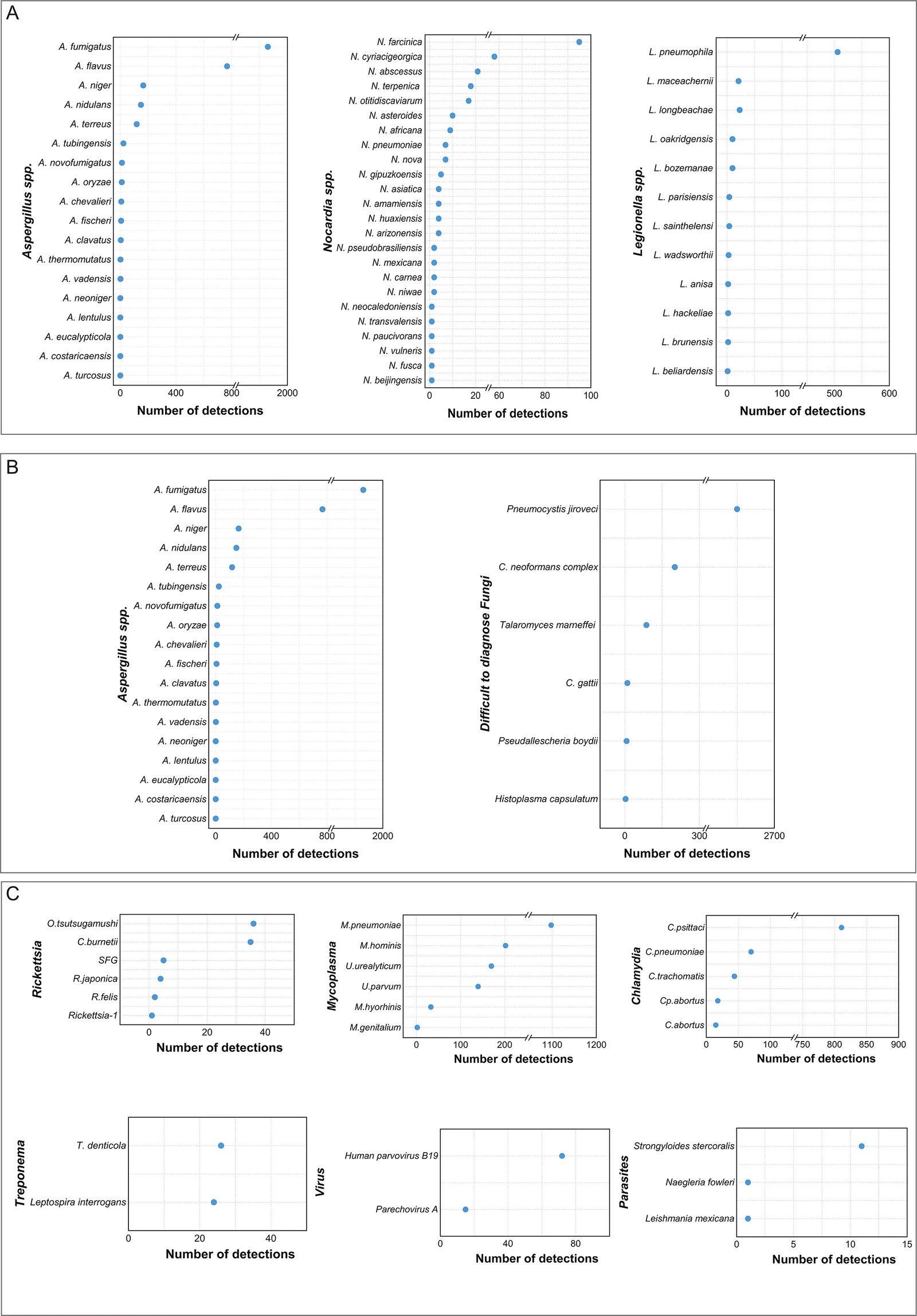
Frequency distributions of several difficult-to-detect pathogens. (**A**) Frequency distributions of several difficult-to-diagnose bacteria, including *Mycobacterium* spp., *Legionella* spp., and *Nocardia* spp. (**B**) Frequency distributions of several difficult-to-detect fungi, including *Aspergillus* spp. and some other difficult-to-diagnose fungi. (**C**) Frequency distributions of several difficult-to-detect uncommon pathogens, including *Rickettsia, Mycoplasma*, *Treponema,* parasites, Chlamydia, and viruses. Data source from April 2019 to May 2025.

Among all 30,819 samples, mNGS detected a total of 109 pathogens that are clinically challenging to identify by conventional microbiological tests. The distribution of the 2,902 Mycobacteria spp. was shown in Fig. 6A, with the *M. tuberculosis* complex being the dominant species within the genus (5.87%), followed by *Mycobacterium abscessus* (0.95%), *Mycobacterium chelonae* (0.89%), and *Mycobacterium avium* complex (0.85%). A similar analysis was conducted for other bacterial genera, including Legionella (1.91%) and Nocardia (0.90%) (Fig. 6A). We also identified *Corynebacterium diphtheriae* in 27 cases. As shown in Fig. 6B, the predominant fungal genus was Aspergillus (10.18%), along with some clinically relevant fungi, such as *P. jirovecii*, Cryptococcus, and *Talaromyces marneffei*. In addition, we identified pathogens for which traditional tests may be lacking, including Mycoplasma (5.34%), Chlamydia (3.11%), Rickettsia (0.27%), Treponema (0.16%), and parasites (0.04%) (Fig. 6C). Of note, human Parvovirus B19 and human Parechovirus A were also detected in our data set, accounting for 0.23% and 0.05% of cases, respectively (Fig. 6C).

### Changes in the pathogen spectrum before, during, and after the COVID-19 pandemic

**Fig 7:**
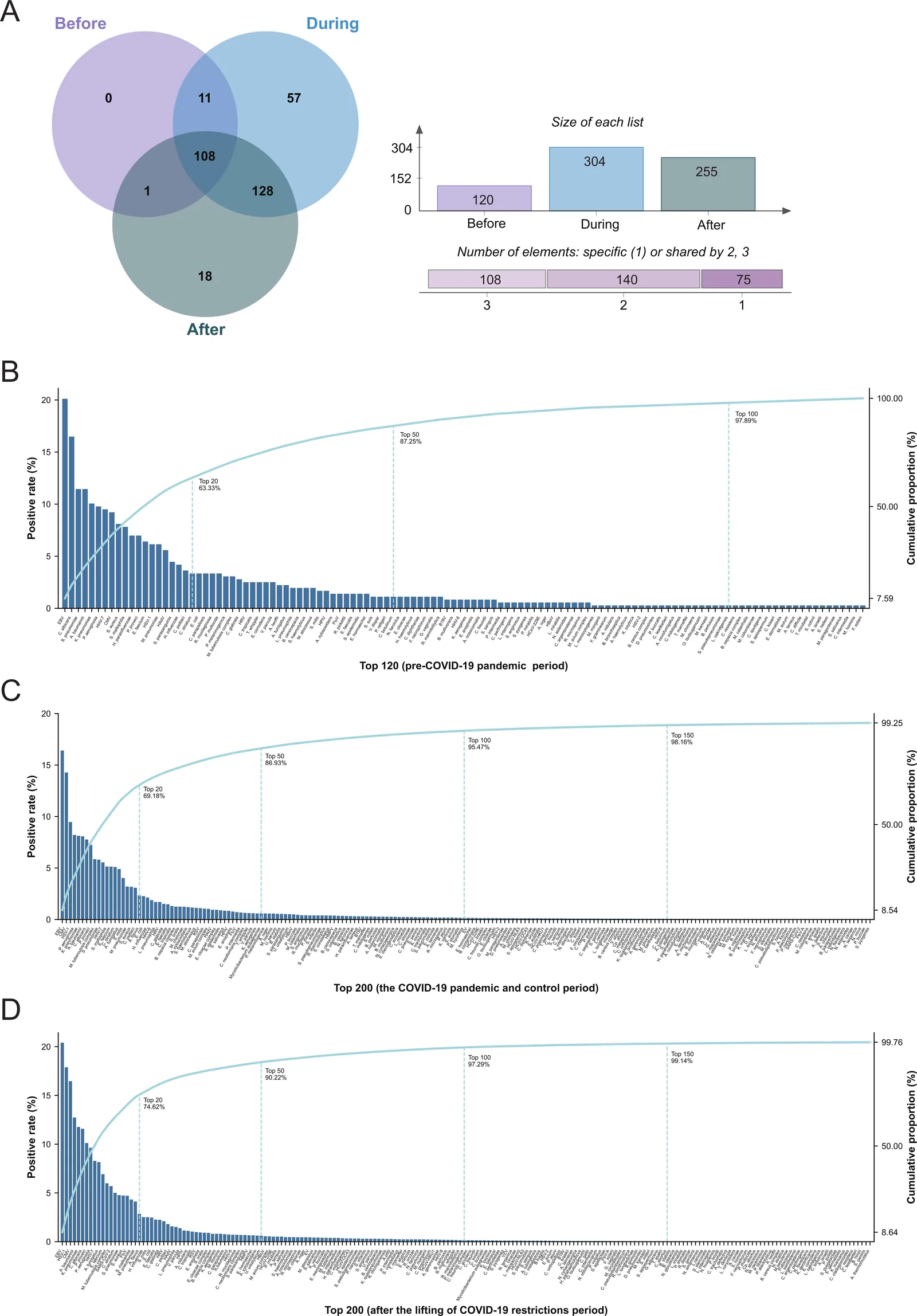
Comparison of the pathogen spectrum before, during, and after the COVID-19 control policy. (**A**) A Venn diagram illustrating the number of unique and shared pathogen species detected before, during, and after COVID-19. (**B-D**) Pareto charts showing the positive detection rates of the top 120–200 pathogens identified (**B**) before, (**C**) during, and (**D**) after the COVID-19 control policy. The cumulative proportion curve in each chart indicates the contribution of the top 20, 50, 100, 150, and 200 pathogens to the total detection rate. If the number of pathogens is less than 200, it shall be calculated based on the actual quantity.

To explore temporal changes, we compared three periods: pre-pandemic (1 April to 31 December 2019; phase 1; *n* = 358), pandemic and control (1 January 2020 to 7 December 2022; phase 2; *n* = 19,417), and post-restriction (8 December 2022 to 31 May 2025; phase 3; *n* = 11,044). We identified 120, 304, and 255 taxa, respectively (Fig. 7). Fifty-seven taxa were observed only in phase 2 and 18 only in phase 3; all had an average detection rate <0.05% (Tables S2 and S3). Table 2 reports Wilson 95% CIs and phase 3 versus phase 1 absolute risk differences for representative taxa. Influenza virus decreased from 1.40% (95% CI, 0.60%–3.23%) in phase 1 to 0.71% (0.60%–0.84%) in phase 2 and increased to 4.72% (4.34%–5.13%) in phase 3; the phase 3 versus phase 1 risk difference was 3.32 percentage points (95% CI, 2.04–4.60). By contrast, the CIs for several apparent U-shaped patterns overlapped substantially with the imprecise phase 1 estimate. Persistent decreases were more evident for *S. pneumoniae* (11.45% to 5.81% to 4.78%; phase 3 versus phase 1 risk difference, −6.67 percentage points [95% CI, −9.99 to −3.35]) than for *C. psittaci* (3.63% to 3.08% to 1.81%; risk difference, −1.82 percentage points [95% CI, −3.77 to 0.13]). *P. jirovecii*, HSV-1, and CMV were more frequently detected in phase 3 than phase 1 (Table 2). Because phase 1 was small and covered only 9 months, these unadjusted period contrasts are descriptive and may reflect sampling mix, referral patterns, specimen type, age, clinical department, and healthcare practice in addition to pandemic-related transmission changes.

**TABLE 2:** Detection proportions and 95% confidence intervals for representative taxa across the three pandemic-related periods^*a*^

Taxon	Phase 1, % (95% CI)	Phase 2, % (95% CI)	Phase 3, % (95% CI)	Phase 3 versus phase 1 RD, pp (95% CI)	Interpretation
Influenza virus	1.40 (0.60–3.23)	0.71 (0.60–0.84)	4.72 (4.34–5.13)	3.32 (2.04 to 4.60)	Increase
Human rhinovirus	2.51 (1.33–4.71)	1.70 (1.53–1.89)	2.10 (1.85–2.39)	−0.41 (−2.06 to 1.23)	Uncertain
*A. baumannii*	11.45 (8.56–15.17)	8.08 (7.71–8.47)	12.74 (12.13–13.37)	1.29 (−2.07 to 4.64)	Uncertain
*K. pneumoniae*	10.06 (7.35–13.61)	8.13 (7.76–8.52)	11.77 (11.18–12.39)	1.72 (−1.46 to 4.89)	Uncertain
*P. aeruginosa*	9.78 (7.11–13.29)	8.21 (7.83–8.60)	9.63 (9.10–10.20)	−0.14 (−3.27 to 2.98)	Uncertain
*C. albicans*	16.48 (13.00–20.68)	7.77 (7.40–8.16)	11.58 (11.00–12.19)	−4.90 (−8.79 to −1.01)	Lower
*C. glabrata*	2.79 (1.52–5.06)	1.52 (1.36–1.70)	2.25 (1.99–2.54)	−0.55 (−2.28 to 1.18)	Uncertain
*P. jirovecii*	6.98 (4.77–10.11)	Not shown	10.12 (9.57–10.70)	3.14 (0.44 to 5.84)	Increase
HSV-1	6.15 (4.09–9.13)	Not shown	17.89 (17.19–18.62)	11.75 (9.16 to 14.34)	Increase
CMV	9.22 (6.64–12.66)	Not shown	16.47 (15.79–17.17)	7.25 (4.18 to 10.33)	Increase
*S. pneumoniae*	11.45 (8.56–15.17)	5.81 (5.49–6.15)	4.78 (4.40–5.19)	−6.67 (−9.99 to −3.35)	Decrease
*C. psittaci*	3.63 (2.13–6.11)	3.08 (2.85–3.33)	1.81 (1.58–2.08)	−1.82 (−3.77 to 0.13)	Uncertain

^*a*^ Values are based on the period-specific denominators (358, 19,417, and 11,044). RD, absolute risk difference; pp, percentage points. “Uncertain” denotes a risk-difference CI including zero. Percentages reported in the archived analysis were used to reconstruct integer counts by nearest rounding. Estimates may differ minimally from analyses of the source database.

## DISCUSSION

In this nationwide retrospective analysis of mNGS reports from hospitalized patients with LRTIs, we observed broad taxonomic diversity and substantial variation in organism detection by age, specimen type, season, geography, clinical department, and pandemic-related period. These data describe laboratory detections rather than etiologically confirmed infections. They may inform hypothesis generation, surveillance priorities, and the design of targeted panels, but they should not be interpreted as population prevalence estimates because mNGS ordering was clinician-selected and contributing hospitals and specimen mixes varied over time.

Co-detections were common among pediatric patients, older adults, and ICU patients. *S. pneumoniae* with *S. mitis* and CMV with *P. jirovecii* were frequent pairs. Because *S. pneumoniae* and *S. mitis* share extensive genomic similarity, some species-level assignments may reflect misclassification. CMV and *P. jirovecii* are also frequently detected together in immunocompromised populations (23, 24). Importantly, mNGS establishes the presence of nucleic acid, not microbial viability, tissue invasion, or causality. We therefore use “co-detection” throughout for two or more organisms reported from the same specimen and reserve “co-infection” for clinically adjudicated disease. The available report database lacked systematic adjudication, conventional-test comparators, antimicrobial changes, or outcomes, so the proportion of co-detections representing true polymicrobial infection cannot be determined.

Compared to sputum and throat swabs, BALF can better represent the microbial profile of the lower respiratory tract (25). In our study, BALF accounted for 67.32% of all samples. However, BALF is obtained through invasive procedures, and our data showed that sputum could be an alternative when bronchoscopy was not feasible. The most detected bacteria were *A. baumannii*, *K. pneumoniae,* and *P. aeruginosa*, which are opportunistic pathogens that could cause acute or chronic respiratory infections in patients over 60 years, as well as *S. pneumoniae*, which is known to cause pneumonia in children (<5 years). Moreover, respiratory viruses, such as HRV, RSV, HAdV, and HPIV, were more likely to be detected in children. Our data also revealed a high prevalence of herpesviruses in respiratory specimens, including EBV, CMV, and HSV-1, which have also been reported by other studies (26–29). The presence of herpesviruses may indicate latent infection, reactivation, or active infections, all of which could pose a significant threat to immunocompromised individuals. Recently, Fabienne Läderach et al. reported that EBV infection can cause multiple sclerosis by inducing CNS homing of B cells, attracting inflammatory T cells (30). Given the fact that EBV was the most prevalent pathogen in respiratory samples in our study, its potential impact on human health warrants special attention.

Being consistent with previous reports, we found that *A. baumannii* was more frequently detected in the northern regions in winter, while *P. aeruginosa* was more prevalent in the southern regions in summer (31, 32). In addition, *C. psittaci*, a pathogen lacking routine diagnostic tools, was more common in southern China (particularly in Jiangxi, Jiangsu, and Zhejiang provinces) (33–35), and peaked in the fall and winter. A previous mNGS study on atypical pulmonary infections in 14 provinces of China reported a 2.1% overall detection rate of *C. psittaci*, with high occurrence in south-central and southwestern regions during winter and spring (36). The distribution and prevalence of *C. psittaci* are likely related to meteorological factors, occupation, diet, and living habits (37, 38).

We discovered a higher prevalence of herpesviruses in southern regions than in the north. Southern China is recognized as an endemic region for EBV-associated nasopharyngeal carcinoma, and the seropositivity rate of antigens expressed during the latent and lysogenic infection stages of EBV is significantly higher in southern China (37,38). Similarly, CMV and HSV-1 were more common in southern Chinese cities (e.g., Wenzhou, Zhejiang; Wuhan, Hubei) based on the results of serum ELISA, PCR, and other methods (39, 40). In contrast, respiratory viruses were more frequently detected in the North. This is likely due to a greater proportion of young children (<5 years) and the pharyngeal swabs in the north in our data.

A key advantage of mNGS is its ability to detect pathogens that are difficult to identify by conventional methods. Here, we detected 109 pathogens that are clinically difficult to identify through conventional microbiological tests, which indicates that mNGS can serve as a complementary diagnostic tool to conventional microbiological tests. Given the diversity of pathogens identified, we advocated the utility of unbiased diagnostic approaches in the epidemiological surveillance of respiratory infections for a better understanding of potential causal pathogens that were previously overlooked or unanticipated. Despite being technically more complex and costly than conventional microbiological tests, mNGS is hypothesis-free, culture-independent, and less influenced by prior antibiotic exposure. With appropriate patient selection, sample handling, and data interpretation, mNGS could become a promising technology for precision diagnosis, guiding clinical interventions and targeted antibiotic administration.

The period analysis identified several contrasting patterns. Influenza virus detection was lower during the pandemic/control period and higher after restrictions were lifted, consistent with altered person-to-person transmission under non-pharmaceutical interventions (41). Some hospital-associated organisms and Candida species showed apparent U-shaped patterns, whereas *S. pneumoniae* declined across all three periods. HSV-1, CMV, and *P. jirovecii* were more frequently detected after restrictions were lifted. Potential mechanisms include changes in healthcare utilization, referral thresholds, specimen type, antimicrobial exposure, age and immune-status mix, infection-control practice, viral circulation, and post-viral immune perturbation (42, 43). However, the study was not designed to attribute these changes to COVID-19 infection or policy. The short, small phase 1 sample produced wide CIs, and neither patient-level SARS-CoV-2 history nor longitudinal immune measurements were available. These trends should therefore be viewed as descriptive surveillance signals requiring confirmation in prospectively standardized cohorts.

This study has several limitations. It was retrospective and based on clinician-selected send-out testing rather than systematic population sampling. Clinical covariates, conventional microbiology, antimicrobial exposure, adjudicated diagnoses, and outcomes were incomplete. Consequently, diagnostic accuracy, treatment impact, and clinical co-infection could not be assessed, and representative case or outcome analyses were not validly supported. The hospital, department, age, and specimen composition may have differed across periods and regions. Phase 1 was particularly small and covered only 9 months, limiting precision and comparability. Peripheral-blood detections may reflect bloodstream involvement, translocation, or latent/reactivated viruses rather than a pulmonary focus. The pus source was incompletely specified. Reference-database content, reagent background, taxonomic similarity, and the report-level exclusion filter could produce false-positive, false-negative, or misclassified findings. Finally, multiple exploratory subgroup comparisons increase the risk of chance findings despite effect-size reporting and false-discovery-rate procedures. Subject to these limitations, the data set defines the breadth of organisms reported by mNGS in routine LRTI practice and provides a resource for generating surveillance hypotheses and prioritizing targets for future prospectively validated tNGS or multiplex-NAAT panels.

## Data Availability

The mNGS assay used in this study is a proprietary laboratory-developed test of Matridx Biotechnology Co., Ltd. Raw sequencing data are not publicly available because of patient privacy considerations and the regulations of the People’s Republic of China on the administration of human genetic resources (2019). De-identified summary data, including patient sex, age, hospital, sampling date, specimen type, symptoms, and preliminary diagnosis when available, and reported microorganisms, are provided in the manuscript and supplemental materials.

## SUPPLEMENTAL MATERIAL

Table S1 spectrum.01746-26-s0001.pdf A total of 323 pathogens were identified in this study.

Table S2 spectrum.01746-26-s0002.pdf The pathogens appear only in the COVID-19 pandemic and control period.

Table S3 spectrum.01746-26-s0003.pdf The pathogens only appear in the period after the lifting of COVID-19 restrictions.

ASM does not own the copyrights to Supplemental Material that may be linked to, or accessed through, an article. The authors have granted ASM a non-exclusive, world-wide license to publish the Supplemental Material files. Please contact the corresponding author directly for reuse.
