# Supplementary material for: A nationwide study of pathogens in hospitalized patients with lower respiratory tract infections by metagenomic next-generation sequencing
Source: Microbiol Spectr. 2026 Sep 23;14(11):e01746-26. doi: 10.1128/spectrum.01746-26 (PMC13729220; doi:10.1128/spectrum.01746-26)
Supplement: Supplementary file 1 [file spectrum.01746-26-s0001.pdf]

Supplementary Table 1. A total of 323 pathogens

NO.	Pathogens	Abbreviation	Pathogen classification	Number	Pathogen positive rate (%)
1	Epstein-Barr virus	EBV	Virus	5513	17·89
2	Cytomegalovirus	CMV	Virus	4625	15·01
3	Human alphaherpesvirus 1	HSV-1	Virus	3837	12·45
4	*Acinetobacter baumannii*	*A • baumannii*	Bacterium	3016	9·79
5	*Klebsiella pneumoniae*	*K • pneumoniae*	Bacterium	2914	9·46
6	*Candida albicans*	*C • albicans*	Fungus	2847	9·24
7	*Pseudomonas aeruginosa*	*P • aeruginosa*	Bacterium	2694	8·74
8	*Pneumocystis jiroveci*	*P • jiroveci*	Fungus	2550	8·27
9	Human betaherpesvirus 7	HHV-7	Virus	2028	6·58
10	*Aspergillus fumigatus*	*A • fumigatus*	Fungus	1860	6·04
11	*Mycobacterium tuberculosis complex*	*M • tuberculosis complex*	Bacterium	1809	5·87
12	*Enterococcus faecium*	*E • faecium*	Bacterium	1786	5·8
13	*Streptococcus pneumoniae*	*S • pneumoniae*	Bacterium	1697	5·51
14	*Corynebacterium striatum*	*C • striatum*	Bacterium	1555	5·05
15	*Stenotrophomonas maltophilia*	*S • maltophilia*	Bacterium	1506	4·89
16	*Staphylococcus aureus*	*S • aureus*	Bacterium	1167	3·79
17	*Mycoplasma pneumoniae*	*M • pneumoniae*	Mycoplasma	1098	3·56
18	*Streptococcus mitis*	*S • mitis*	Bacterium	1067	3·46
19	*Chlamydia psittaci*	*C • psittaci*	Chlamydia	811	2·63
20	*Aspergillus flavus*	*A • flavus*	Fungus	769	2·5
21	*Escherichia coli*	*E • coli*	Bacterium	726	2·36
22	*Haemophilus influenzae*	*H • influenzae*	Bacterium	709	2·3
23	Influenza virus	FluV	Virus	666	2·16
24	severe acute respiratory syndrome coronavirus 2	SARS-CoV-2	Virus	637	2·07
25	Human rhinovirus	HRV	Virus	571	1·85
26	*Candida glabrata*	*C • glabrata*	Fungus	554	1·8
27	*Enterococcus faecalis*	*E • faecalis*	Bacterium	544	1·77
28	*Legionella pneumophila*	*L • pneumophila*	Bacterium	506	1·64
29	Human betaherpesvirus 6	HHV-6	Virus	447	1·45
30	*Candida tropicalis*	*C • tropicalis*	Fungus	378	1·23
31	Human adenovirus	HAdV	Virus	377	1·22
32	*Streptococcus oralis*	*S • oralis*	Bacterium	344	1·12
33	*Burkholderia cepacia complex*	*B • cepaciacomplex*	Bacterium	334	1·08
34	Respiratory syncytial virus	RSV	Virus	323	1·05
35	*Achromobacter xylosoxidans*	*A • xylosoxidans*	Bacterium	308	1
36	*Candida parapsilosis*	*C • parapsilosis*	Fungus	301	0·98
37	Human parainfluenza virus	HPIV	Virus	297	0·96
38	*Mycobacterium chelonae*	*M • chelonae*	Bacterium	292	0·95
39	*Staphylococcus haemolyticus*	*S • haemolyticus*	Bacterium	285	0·92
40	*Enterobacter cloacae complex*	*E • cloacaecomplex*	Bacterium	283	0·92
41	*Veillonella parvula*	*V • parvula*	Bacterium	282	0·92
42	*Mycobacterium abscessus*	*M • abscessus*	Bacterium	273	0·89
43	*Mycobacterium avium complex*	*M • aviumcomplex*	Bacterium	263	0·85
44	*Elizabethkingia anophelis*	*E • anophelis*	Bacterium	258	0·84
45	Staphylococcus epidermidis	S • epidermidis	Bacterium	215	0·7
46	Streptococcus salivarius	S • salivarius	Bacterium	214	0·69
47	*Klebsiella aerogenes*	*K • aerogenes*	Bacterium	207	0·67
48	*Mycoplasma hominis*	*M • hominis*	Mycoplasma	202	0·66
49	*Cryptococcus neoformans species complex*	*C • neoformanscomplex*	Fungus	201	0·65
50	*Streptococcus parasanguinis*	*S • parasanguinis*	Bacterium	195	0·63
51	*Ureaplasma urealyticum*	*U • urealyticum*	Mycoplasma	170	0·55
52	*Rothia mucilaginosa*	*R • mucilaginosa*	Bacterium	168	0·55
53	*Aspergillus niger*	*A • niger*	Fungus	165	0·54

54	*Prevotella intermedia*	*P • intermedia*	Bacterium	164	0·53
55	Human bocavirus	HBoV	Virus	161	0·52
56	*Aspergillus nidulans*	*A • nidulans*	Fungus	149	0·48
57	*Tropheryma whipplei*	*T • whipplei*	Bacterium	148	0·48
58	Human metapneumovirus	HMPV	Virus	147	0·48
59	*Prevotella melaninogenica*	*P • melaninogenica*	Bacterium	142	0·46
60	*Ureaplasma parvum*	*U • parvum*	Mycoplasma	140	0·45
61	*Proteus mirabilis*	*P • mirabilis*	Bacterium	137	0·44
62	*Prevotella buccalis*	*P • buccalis*	Bacterium	134	0·43
63	*Mycolicibacterium aubagnense*	*M • aubagnense*	Bacterium	129	0·42
64	*Haemophilus parainfluenzae*	*H • parainfluenzae*	Bacterium	127	0·41
65	*Moraxella catarrhalis*	*M • catarrhalis*	Bacterium	123	0·4
66	*Aspergillus terreus*	*A • terreus*	Fungus	118	0·38
67	*Streptococcus anginosus*	*S • anginosus*	Bacterium	115	0·37
68	*Streptococcus pseudopneumoniae*	*S • pseudopneumoniae*	Bacterium	114	0·37
69	*Streptococcus intermedius*	*S • intermedius*	Bacterium	111	0·36
70	*Streptococcus infantis*	*S • infantis*	Bacterium	111	0·36
71	*Enterobacter hormaechei*	*E • hormaechei*	Bacterium	110	0·36
72	*Burkholderia cenocepacia*	*B • cenocepacia*	Bacterium	110	0·36
73	*Burkholderia stabilis*	*B • stabilis*	Bacterium	109	0·35
74	*Acinetobacter nosocomialis*	*A • nosocomialis*	Bacterium	108	0·35
75	*Streptococcus constellatus*	*S • constellatus*	Bacterium	104	0·34
76	*Ralstonia mannitolilytica*	*R • mannitolilytica*	Bacterium	98	0·32
77	*Chryseobacterium indologenes*	*C • indologenes*	Bacterium	97	0·31
78	*Nocardia farcinica*	*N • farcinica*	Bacterium	95	0·31
79	*Elizabethkingia meningoseptica*	*E • meningoseptica*	Bacterium	90	0·29
80	*Rhizopus microsporus*	*R • microsporus*	Fungus	89	0·29
81	*Talaromyces marneffei*	*T • marneffei*	Fungus	87	0·28
82	Human Coronavirus 229E	HCoV-229E	Virus	86	0·28
83	Acinetobacter baumannii-calcoaceticus complex	ABC	Bacterium	85	0·28
84	Enterovirus	EV	Virus	82	0·27
85	*Klebsiella variicola*	*K • variicola*	Bacterium	81	0·26
86	*Morganella morganii*	*M • morganii*	Bacterium	78	0·25
87	*Bordetella pertussis*	*B • pertussis*	Bacterium	76	0·25
88	*Burkholderia multivorans*	*B • multivorans*	Bacterium	75	0·24
89	Human parvovirus B19	B19V	Virus	72	0·23
90	*Burkholderia cepacia*	*B • cepacia*	Bacterium	71	0·23
91	*Chlamydia pneumoniae*	*C • pneumoniae*	Chlamydia	70	0·23
92	*Elizabethkingia miricola*	*E • miricola*	Bacterium	64	0·21
93	*Corynebacterium accolens*	*C • accolens*	Bacterium	63	0·2
94	*Human Coronavirus OC43*	*HCoV-OC43*	Virus	61	0·2
95	*Streptococcus milleri*	*S • milleri*	Bacterium	60	0·19
96	*Nocardia cyriacigeorgica*	*N • cyriacigeorgica*	Bacterium	58	0·19
97	*Klebsiella oxytoca*	*K • oxytoca*	Bacterium	56	0·18
98	*Prevotella salivae*	*P • salivae*	Bacterium	55	0·18
99	*Lautropia mirabilis*	*L • mirabilis*	Bacterium	53	0·17
100	*Rhizopus oryzae*	*R • oryzae*	Fungus	49	0·16
101	*Sphingomonas paucimobilis*	*S • paucimobilis*	Bacterium	48	0·16
102	*Corynebacterium propinquum*	*C • propinquum*	Bacterium	46	0·15
103	Human coronavirus HKU1	HCoV-HKU1	Virus	46	0·15
104	*Klebsiella quasipneumoniae*	*K • quasipneumoniae*	Bacterium	46	0·15
105	*Prevotella jejuni*	*P • jejuni*	Bacterium	45	0·15
106	*Enterococcus avium*	*E • avium*	Bacterium	45	0·15
107	*Campylobacter showae*	*C • showae*	Bacterium	45	0·15
108	*Citrobacter freundii complex*	*C • freundiicomplex*	Bacterium	44	0·14
109	*Chlamydia trachomatis*	*C • trachomatis*	Chlamydia	44	0·14

110	*Alloprevotella tannerae*	*A • tannerae*	Bacterium	44	0·14
111	*Streptococcus peroris*	*S • peroris*	Bacterium	43	0·14
112	*Enterobacter cloacae*	*E • cloacae*	Bacterium	43	0·14
113	*Veillonella atypica*	*V • atypica*	Bacterium	42	0·14
114	*Prevotella nanceiensis*	*P • nanceiensis*	Bacterium	41	0·13
115	*Citrobacter freundii*	*C • freundii*	Bacterium	41	0·13
116	*Klebsiella michiganensis*	*K • michiganensis*	Bacterium	41	0·13
117	*Peptostreptococcus stomatis*	*P • stomatis*	Bacterium	38	0·12
118	*Actinomyces gerencseriae*	*A • gerencseriae*	Bacterium	37	0·12
119	Human alphaherpesvirus 2	HSV-2	Virus	37	0·12
120	*Citrobacter braakii*	*C • braakii*	Bacterium	36	0·12
121	*Orientia tsutsugamushi*	*O • tsutsugamushi*	Rickettsia	36	0·12
122	Human gammaherpesvirus 8	HHV-8	Virus	35	0·11
123	*Streptococcus sanguis*	*S • sanguis*	Bacterium	35	0·11
124	Human Coronavirus NL63	HCoV-NL63	Virus	35	0·11
125	*Citrobacter koseri*	*C • koseri*	Bacterium	35	0·11
126	*Coxiella burnetii*	*C • burnetii*	Rickettsia	35	0·11
127	*Mycoplasma hyorhinis*	*M • hyorhinis*	Mycoplasma	33	0·11
128	*Dialister pneumosintes*	*D • pneumosintes*	Bacterium	32	0·1
129	*Acinetobacter pittii*	*A • pittii*	Bacterium	31	0·1
130	*Burkholderia contaminans*	*B • contaminans*	Bacterium	30	0·1
131	*Scedosporium apiospermum*	*S • apiospermum*	Fungus	30	0·1
132	*Corynebacterium diphtheriae*	*C • diphtheriae*	Bacterium	27	0·09
133	*Treponema denticola*	*T • denticola*	Spirochaeta	26	0·08
134	*Mycobacterium paragordonae*	*M • paragordonae*	Bacterium	26	0·08
135	*Candida intermedia*	*C • intermedia*	Fungus	26	0·08
136	*Candida metapsilosis*	*C • metapsilosis*	Fungus	26	0·08
137	*Cunninghamella bertholletiae*	*C • bertholletiae*	Fungus	25	0·08
138	*Mycobacterium chimaera*	*M • chimaera*	Bacterium	25	0·08
139	*Enterocytozoon bieneusi*	*E • bieneusi*	Fungus	24	0·08
140	*Leptospira interrogans*	*L • interrogans*	Spirochaeta	24	0·08
141	*Veillonella dispar*	*V • dispar*	Bacterium	23	0·07
142	*Legionella longbeachae*	*L • longbeachae*	Bacterium	23	0·07
143	*Aspergillus tubingensis*	*A • tubingensis*	Fungus	23	0·07
144	*Candida orthopsilosis*	*C • orthopsilosis*	Fungus	23	0·07
145	*Streptococcus australis*	*S • australis*	Bacterium	22	0·07
146	*Legionella maceachernii*	*L • maceachernii*	Bacterium	21	0·07
147	*Nocardia abscessus*	*N • abscessus*	Bacterium	21	0·07
148	*Eikenella corrodens*	*E • corrodens*	Bacterium	21	0·07
149	*Raoultella ornithinolytica*	*R • ornithinolytica*	Bacterium	21	0·07
150	*Streptococcus agalactiae*	*S • agalactiae*	Bacterium	20	0·06
151	*Lichtheimia ramosa*	*L • ramosa*	Fungus	20	0·06
152	*Haemophilus parahaemolyticus*	*H • parahaemolyticus*	Bacterium	20	0·06
153	*Streptococcus gordonii*	*S • gordonii*	Bacterium	20	0·06
154	*Streptococcus pyogenes*	*S • pyogenes*	Bacterium	20	0·06
155	*Bacillus cereus complex*	*B • cereuscomplex*	Bacterium	19	0·06
156	*Clostridium botulinum*	*C • botulinum*	Bacterium	19	0·06
157	*Pseudomonas putida*	*P • putida*	Bacterium	19	0·06
158	*Fusarium oxysporum*	*F • oxysporum*	Fungus	19	0·06
159	*Chlamydophila abortus*	*Cp • abortus*	Chlamydia	18	0·06
160	*Corynebacterium segmentosum*	*C • segmentosum*	Bacterium	18	0·06
161	*Tatlockia micdadei*	*T • micdadei*	Bacterium	18	0·06
162	*Pasteurella multocida*	*P • multocida*	Bacterium	18	0·06
163	*Nocardia terpenica*	*N • terpenica*	Bacterium	18	0·06
164	*Phocaeicola vulgatus*	*P • vulgatus*	Bacterium	18	0·06
165	*Gardnerella vaginalis*	*G • vaginalis*	Bacterium	17	0·06

166	*Nocardia otitidiscaviarum*	*N • otitidiscaviarum*	Bacterium	17	0·06
167	*Mycobacterium kansasii*	*M • kansasii*	Bacterium	16	0·05
168	*Fusobacterium necrophorum*	*F • necrophorum*	Bacterium	15	0·05
169	Parechovirus A	PeV-A	Virus	15	0·05
170	*Chlamydia abortus*	*C • abortus*	Chlamydia	15	0·05
171	Varicella-zoster virus	VZV	Virus	15	0·05
172	*Granulicatella adiacens*	*G • adiacens*	Bacterium	14	0·05
173	*Streptococcus vestibularis*	*S • vestibularis*	Bacterium	14	0·05
174	*Corynebacterium pseudodiphtheriticum*	*C • pseudodiphtheriticum*	Bacterium	14	0·05
175	*Mycolicibacterium phocaicum*	*M • phocaicum*	Bacterium	13	0·04
176	*Aspergillus novofumigatus*	*A • novofumigatus*	Fungus	13	0·04
177	*Trichosporon asahii*	*T • asahii*	Fungus	12	0·04
178	*Acinetobacter lwoffii*	*A • lwoffii*	Bacterium	12	0·04
179	*Aspergillus oryzae*	*A • oryzae*	Fungus	11	0·04
180	*Rhizopus delemar*	*R • delemar*	Fungus	11	0·04
181	*Abiotrophia defectiva*	*A • defectiva*	Bacterium	11	0·04
182	*Strongyloides stercoralis*	*S • stercoralis*	Parasite	11	0·04
183	*Staphylococcus hominis*	*S • hominis*	Bacterium	11	0·04
184	*Clostridium perfringens*	*C • perfringens*	Bacterium	11	0·04
185	*Bacteroides thetaiotaomicron*	*B • thetaiotaomicron*	Bacterium	11	0·04
186	*Achromobacter denitrificans*	*A • denitrificans*	Bacterium	11	0·04
187	*Ralstonia pickettii*	*R • pickettii*	Bacterium	11	0·04
188	*Legionella bozemanae*	*L • bozemanae*	Bacterium	10	0·03
189	*Pseudoramibacter alactolyticus*	*P • alactolyticus*	Bacterium	10	0·03
190	*Corynebacterium resistens*	*C • resistens*	Bacterium	10	0·03
191	*Bordetella hinzii*	*B • hinzii*	Bacterium	10	0·03
192	*Mucor racemosus*	*M • racemosus*	Fungus	10	0·03
193	*Kluyveromyces marxianus*	*K • marxianus*	Fungus	10	0·03
194	*Bordetella bronchiseptica*	*B • bronchiseptica*	Bacterium	10	0·03
195	*Corynebacterium argentoratense*	*C • argentoratense*	Bacterium	10	0·03
196	*Legionella oakridgensis*	*L • oakridgensis*	Bacterium	10	0·03
197	*Mycolicibacterium mucogenicum*	*M • mucogenicum*	Bacterium	10	0·03
198	*Cryptococcus gattii*	*C • gattii*	Fungus	10	0·03
199	*Nocardia asteroides*	*N • asteroides*	Bacterium	10	0·03
200	*Aeromonas caviae*	*A • caviae*	Bacterium	10	0·03
201	*Mycolicibacterium fortuitum*	*M • fortuitum*	Bacterium	9	0·03
202	*Dolosigranulum pigrum*	*D • pigrum*	Bacterium	9	0·03
203	*Nocardia africana*	*N • africana*	Bacterium	9	0·03
204	*Mycobacterium colombiense*	*M • colombiense*	Bacterium	9	0·03
205	*Neisseria flavescens*	*N • flavescens*	Bacterium	9	0·03
206	*Haemophilus haemolyticus*	*H • haemolyticus*	Bacterium	9	0·03
207	*Prevotella nigrescens*	*P • nigrescens*	Bacterium	8	0·03
208	*Fusarium solani*	*F • solani*	Fungus	8	0·03
209	*Providencia rettgeri*	*P • rettgeri*	Bacterium	8	0·03
210	*Aspergillus chevalieri*	*A • chevalieri*	Fungus	8	0·03
211	*Kingella denitrificans*	*K • denitrificans*	Bacterium	8	0·03
212	*Clostridioides difficile*	*C • difficile*	Bacterium	8	0·03
213	*Mucor circinelloides*	*M • circinelloides*	Fungus	8	0·03
214	*Prevotella enoeca*	*P • enoeca*	Bacterium	8	0·03
215	*Bordetella parapertussis*	*B • parapertussis*	Bacterium	7	0·02
216	*Pseudallescheria boydii*	*P • boydii*	Fungus	7	0·02
217	*Prevotella buccae*	*P • buccae*	Bacterium	7	0·02
218	*Lichtheimia corymbifera*	*L • corymbifera*	Fungus	7	0·02
219	*Aspergillus fischeri*	*A • fischeri*	Fungus	7	0·02
220	*Nocardia pneumoniae*	*N • pneumoniae*	Bacterium	7	0·02
221	*Nocardia nova*	*N • nova*	Bacterium	7	0·02

222	*Streptococcus equinus*	*S・ equinus*	Bacterium	7	0·02
223	*Klebsiella grimontii*	*K・ grimontii*	Bacterium	6	0·02
224	*Listeria monocytogenes*	*L・ monocytogenes*	Bacterium	6	0·02
225	*Streptococcus suis*	*S・ suis*	Bacterium	6	0·02
226	*Corynebacterium jeikeium*	*C・ jeikeium*	Bacterium	6	0·02
227	*Capnocytophaga leadbetteri*	*C・ leadbetteri*	Bacterium	6	0·02
228	*Gemella morbillorum*	*G・ morbillorum*	Bacterium	6	0·02
229	*Pseudomonas libanensis*	*P・ libanensis*	Bacterium	6	0·02
230	*Prevotella dentalis*	*P・ dentalis*	Bacterium	6	0·02
231	*Nocardia gipuzkoensis*	*N・ gipuzkoensis*	Bacterium	5	0·02
232	*Actinomyces israelii*	*A・ israelii*	Bacterium	5	0·02
233	*Acinetobacter haemolyticus*	*A・ haemolyticus*	Bacterium	5	0·02
234	Spotted fever group	SFG	Rickettsia	5	0·02
235	*Encephalitozoon hellem*	*E・ hellem*	Fungus	5	0·02
236	*Burkholderia pseudomallei*	*B・ pseudomallei*	Bacterium	5	0·02
237	*Aeromonas veronii*	*A・ veronii*	Bacterium	5	0·02
238	*Chryseobacterium gleum*	*C・ gleum*	Bacterium	5	0·02
239	*Mycobacterium neoaurum*	*M・ neoaurum*	Bacterium	5	0·02
240	*Acinetobacter radioresistens*	*A・ radioresistens*	Bacterium	5	0·02
241	*Staphylococcus cohnii*	*S・ cohnii*	Bacterium	5	0·02
242	*Pseudomonas stutzeri*	*P・ stutzeri*	Bacterium	5	0·02
243	*Nocardia asiatica*	*N・ asiatica*	Bacterium	4	0·01
244	*Escherichia fergusonii*	*E・ fergusonii*	Bacterium	4	0·01
245	*Mycobacterium aurum*	*M・ aurum*	Bacterium	4	0·01
246	*Fusarium fujikuroi*	*F・ fujikuroi*	Fungus	4	0·01
247	*Magnusiomyces capitatus*	*M・ capitatus*	Fungus	4	0·01
248	*Providencia stuartii*	*P・ stuartii*	Bacterium	4	0·01
249	*Rhizopus nigricans*	*R・ nigricans*	Fungus	4	0·01
250	*Cladosporium cladosporioides*	*C・ cladosporioides*	Fungus	4	0·01
251	*Exophiala dermatitidis*	*E・ dermatitidis*	Fungus	4	0·01
252	*Ralstonia insidiosa*	*R・ insidiosa*	Bacterium	4	0·01
253	*Rickettsia japonica*	*R・ japonica*	Rickettsia	4	0·01
254	*Legionella sainthelensi*	*L・ sainthelensi*	Bacterium	4	0·01
255	*Mycobacterium immunogenum*	*M・ immunogenum*	Bacterium	4	0·01
256	*Nocardia huaxiensis*	*N・ huaxiensis*	Bacterium	4	0·01
257	*Aspergillus clavatus*	*A・ clavatus*	Fungus	4	0·01
258	*Corynebacterium aurimucosum*	*C・ aurimucosum*	Bacterium	4	0·01
259	*Fusarium graminearum*	*F・ graminearum*	Fungus	4	0·01
260	*Moraxella nonliquefaciens*	*M・ nonliquefaciens*	Bacterium	4	0·01
261	*Veillonella tobetsuensis*	*V・ tobetsuensis*	Bacterium	4	0·01
262	*Legionella parisiensis*	*L・ parisiensis*	Bacterium	4	0·01
263	*Nocardia amamiensis*	*N・ amamiensis*	Bacterium	4	0·01
264	*Staphylococcus capitis*	*S・ capitis*	Bacterium	4	0·01
265	*Mycobacterium mageritense*	*M・ mageritense*	Bacterium	4	0·01
266	*Nocardia arizonensis*	*N・ arizonensis*	Bacterium	4	0·01
267	*Parabacteroides abscessus*	*P・ abscessus*	Bacterium	3	0·01
268	*Histoplasma capsulatum*	*H・ capsulatum*	Fungus	3	0·01
269	*Mycobacterium seoulense*	*M・ seoulense*	Bacterium	3	0·01
270	*Prevotella multiformis*	*P・ multiformis*	Bacterium	3	0·01
271	*Streptococcus dysgalactiae*	*S・ dysgalactiae*	Bacterium	3	0·01
272	Human T-cell lymphotropic virus type 1	HTLV-1	Virus	3	0·01
273	*Enterococcus raffinosus*	*E・ raffinosus*	Bacterium	3	0·01
274	*Legionella wadsworthii*	*L・ wadsworthii*	Bacterium	3	0·01
275	*Raoultella planticola*	*R・ planticola*	Bacterium	3	0·01
276	*Candida dubliniensis*	*C・ dubliniensis*	Fungus	3	0·01
277	*Capnocytophaga sputigena*	*C・ sputigena*	Bacterium	3	0·01

278	*Bacteroides pyogenes*	*B*・ *pyogenes*	Bacterium	3	0·01
279	*Scedosporium prolificans*	*S*・ *prolificans*	Fungus	3	0·01
280	*Aspergillus lentulus*	*A*・ *lentulus*	Fungus	2	0·01
281	*Nocardia mexicana*	*N*・ *mexicana*	Bacterium	2	0·01
282	*Legionella anisa*	*L*・ *anisa*	Bacterium	2	0·01
283	*Mycoplasma genitalium*	*M*・ *genitalium*	Mycoplasma	2	0·01
284	*Fusobacterium nucleatum*	*F*・ *nucleatum*	Bacterium	2	0·01
285	*Nocardia niwae*	*N*・ *niwae*	Bacterium	2	0·01
286	*Ochrobactrum anthropi*	*O*・ *anthropi*	Bacterium	2	0·01
287	*Peptoniphilus harei*	*P*・ *harei*	Bacterium	2	0·01
288	*Legionella brunensis*	*L*・ *brunensis*	Bacterium	2	0·01
289	*Legionella beliardensis*	*L*・ *beliardensis*	Bacterium	2	0·01
290	*Nocardia carnea*	*N*・ *carnea*	Bacterium	2	0·01
291	*Streptococcus ilei*	*S*・ *ilei*	Bacterium	2	0·01
292	*Mycobacterium iranicum*	*M*・ *iranicum*	Bacterium	2	0·01
293	*Rickettsia felis*	*R*・ *felis*	Rickettsia	2	0·01
294	*Nocardia pseudobrasiliensis*	*N*・ *pseudobrasiliensis*	Bacterium	2	0·01
295	*Aspergillus thermomutatus*	*A*・ *thermomutatus*	Fungus	2	0·01
296	*Aspergillus eucalypticola*	*A*・ *eucalypticola*	Fungus	1	0
297	*Mycobacterium intermedium*	*M*・ *intermedium*	Bacterium	1	0
298	*Nocardia paucivorans*	*N*・ *paucivorans*	Bacterium	1	0
299	*Leishmania mexicana*	*L*・ *mexicana*	Parasite	1	0
300	*Cladosporium sphaerospermum*	*C*・ *sphaerospermum*	Fungus	1	0
301	*Legionella hackeliae*	*L*・ *hackeliae*	Bacterium	1	0
302	*Aspergillus turcosus*	*A*・ *turcosus*	Fungus	1	0
303	*Naegleria fowleri*	*N*・ *fowleri*	Parasite	1	0
304	*Mycobacterium gastri*	*M*・ *gastri*	Bacterium	1	0
305	Torque teno virus	TTV	Virus	1	0
306	*Aureobasidium pullulans*	*A*・ *pullulans*	Fungus	1	0
307	*Nocardia vulneris*	*N*・ *vulneris*	Bacterium	1	0
308	*Rickettsia*	*Rickettsia-1*	Rickettsia	1	0
309	Measles virus	MeV	Virus	1	0
310	*Nocardia beijingensis*	*N*・ *beijingensis*	Bacterium	1	0
311	*Mycobacterium timonense*	*M*・ *timonense*	Bacterium	1	0
312	*Nocardia neocaledoniensis*	*N*・ *neocaledoniensis*	Bacterium	1	0
313	*Mycobacterium tusciae*	*M*・ *tusciae*	Bacterium	1	0
314	*Nocardia fusca*	*N*・ *fusca*	Bacterium	1	0
315	Orf virus	ORFV	Virus	1	0
316	Yersinia frederiksenii	Y・ frederiksenii	Bacterium	1	0
317	*Aspergillus costaricaensis*	*A*・ *costaricaensis*	Fungus	1	0
318	*Aspergillus vadensis*	*A*・ *vadensis*	Fungus	1	0
319	*Micrococcus luteus*	*M*・ *luteus*	Bacterium	1	0
320	*Nocardia transvalensis*	*N*・ *transvalensis*	Bacterium	1	0
321	*Mycobacterium obuense*	*M*・ *obuense*	Bacterium	1	0
322	*Aspergillus neoniger*	*A*・ *neoniger*	Fungus	1	0
323	*Mycobacterium genavense*	*M*・ *genavense*	Bacterium	1	0
