# Supplementary material for: A nationwide study of pathogens in hospitalized patients with lower respiratory tract infections by metagenomic next-generation sequencing
Source: Microbiol Spectr. 2026 Sep 23;14(11):e01746-26. doi: 10.1128/spectrum.01746-26 (PMC13729220; doi:10.1128/spectrum.01746-26)
Supplement: Supplementary file 2 [file spectrum.01746-26-s0002.pdf]

Supplementary Table 2. The pathogens only appear in the COVID-19 pandemic and control period (Phase 2)

Pathogens	Positive number	Positive rate (%)
Mycoplasma hyorhinis	33	0·17
Streptococcus australis	22	0·11
Mycolicibacterium phocaicum	13	0·07
Achromobacter denitrificans	11	0·06
Mycolicibacterium mucogenicum	10	0·05
Mycolicibacterium fortuitum	9	0·05
Prevotella nigrescens	8	0·04
Aspergillus fischeri	7	0·04
Streptococcus equinus	7	0·04
Bordetella parapertussis	7	0·04
Prevotella dentalis	6	0·03
Chryseobacterium gleum	5	0·03
Spotted fever group	5	0·03
Pseudomonas stutzeri	5	0·03
Acinetobacter radioresistens	5	0·03
Aeromonas veronii	5	0·03
Legionella parisiensis	4	0·02
Magnusiomyces capitatus	4	0·02
Aspergillus clavatus	4	0·02
Ralstonia insidiosa	4	0·02
Fusarium fujikuroi	4	0·02
Mycobacterium immunogenum	4	0·02
Mycobacterium mageritense	4	0·02
Corynebacterium aurimucosum	4	0·02
Moraxella nonliquefaciens	4	0·02
Histoplasma capsulatum	3	0·02
Candida dubliniensis	3	0·02
Bacteroides pyogenes	3	0·02
Legionella wadsworthii	3	0·02
Human T-cell lymphotropic virus type 1	3	0·02
Enterococcus raffinosus	3	0·02
Mycoplasma genitalium	2	0·01
Legionella brunensis	2	0·01
Legionella beliardensis	2	0·01
Rickettsia felis	2	0·01
Nocardia niwae	2	0·01
Mycobacterium iranicum	2	0·01
Nocardia paucivorans	1	0·01
Mycobacterium genavense	1	0·01
Legionella hackeliae	1	0·01
Aspergillus turcosus	1	0·01
Mycobacterium gastri	1	0·01
Mycobacterium obuense	1	0·01
Mycobacterium intermedium	1	0·01
Mycobacterium tusciae	1	0·01
Measles virus	1	0·01

Nocardia neocaledoniensis	1	0·01
Orf virus	1	0·01
Aureobasidium pullulans	1	0·01
Leishmania mexicana	1	0·01
Cladosporium sphaerospermum	1	0·01
Yersinia frederiksenii	1	0·01
Aspergillus costaricaensis	1	0·01
Aspergillus neoniger	1	0·01
Aspergillus vadensis	1	0·01
Naegleria fowleri	1	0·01
Aspergillus eucalypticola	1	0·01
