# Supplementary material for: A nationwide study of pathogens in hospitalized patients with lower respiratory tract infections by metagenomic next-generation sequencing
Source: Microbiol Spectr. 2026 Sep 23;14(11):e01746-26. doi: 10.1128/spectrum.01746-26 (PMC13729220; doi:10.1128/spectrum.01746-26)
Supplement: Supplementary file 3 [file spectrum.01746-26-s0003.pdf]

**Supplementary Table 3. The pathogens only appear in after the lifting of COVID-19 restrictions period (Phase 3)**

| Pathogens | Positive number | Positive rate (%) |
|---|---|---|
| *Acinetobacter pittii* | 31 | 0.28 |
| *Bacteroides thetaiotaomicron* | 11 | 0.1 |
| *Klebsiella grimontii* | 6 | 0.05 |
| *Nocardia gipuzkoensis* | 5 | 0.05 |
| *Nocardia amamiensis* | 4 | 0.04 |
| *Nocardia huaxiensis* | 4 | 0.04 |
| *Mycobacterium seoulense* | 3 | 0.03 |
| *Parabacteroides abscessus* | 3 | 0.03 |
| *Aspergillus thermomutatus* | 2 | 0.02 |
| *Fusobacterium nucleatum* | 2 | 0.02 |
| *Micrococcus luteus* | 1 | 0.01 |
| *Nocardia transvalensis* | 1 | 0.01 |
| *Nocardia beijingensis* | 1 | 0.01 |
| *Mycobacterium timonense* | 1 | 0.01 |
| *Nocardia fusca* | 1 | 0.01 |
| *Rickettsia* | 1 | 0.01 |
| *Nocardia vulneris* | 1 | 0.01 |
| *Torque teno virus* | 1 | 0.01 |
